# The Patient Safety Incident Response 5 Years On

**DOI:** 10.1177/17504589251410125

**Published:** 2026-01-26

**Authors:** Rita Couto, John Welch

The Patient Safety Incident Response Framework (or PSIRF) is the national framework underpinning the NHS’s new approach to developing and maintaining effective responses to patient safety incidents. PSIRF was first introduced in August 2022, with the aim of shifting from blaming individuals to a system-based approach to understand and learn from safety incidents. It was put in place to replace the previously used Serious Incident Framework (SIF), as there was general consensus that the SIF did not actively encourage learning or improvement in patient safety. The NHS made it mandatory for all NHS trusts to adhere to PSIRF from Autumn 2023.

PSIRF is designed to ensure that sufficient resources are dedicated to the learning and educational initiatives required to drive improvements in patient safety. Therefore, the processes set out by PSIRF aim to respond to patient safety incidents by encouraging learning from these incidents. Patient safety incidents may include unintended or unexpected events, such as the omission of necessary actions in a healthcare setting. These incidents are not necessarily limited to those that have caused harm to patients, but also include instances where there was the possibility of causing harm, which are often referred to as near misses.

The framework is based on four main principles: (1) ensuring that there is a compassionate and understanding engagement with those affected by any form of patient safety interactions so that a just culture is created, (2) using a system-based approach to learn from both individual patient safety incidents and patterns of patient safety incidents, (3) applying adequate and proportionate responses to patient safety incidents – not every case needs forensic analysis and (4) a focus on continuous improvement of patient safety and the responses to patient safety incidents.

In the same manner that PSIRF replaced the SIF, the Centre for Perioperative Care developed National Safety Standards for Invasive Procedures in 2015, which were then revised and updated in 2023 (NatSSIPs 2), with the aim of keeping up with the increasing complexity of healthcare systems. The foci of NatSSIPs 2 are to improve patient safety, teamwork and efficiency. Similar to PSIRF, these revised standards were put in place to ensure the sharing of learning and best practices to support multidisciplinary teams in delivering safe care. Therefore, in perioperative care, the principles of PSIRF align with those of NatSSIPs 2.

Some of the most common safety issues within perioperative care include miscommunications, wrong site, procedure or patient surgery, leaving instruments inside patients, equipment failures and medication errors (including not checking patients’ allergies). Nevertheless, it has been argued that near misses happen more often in perioperative care than actual safety incidents (Stucky et al 2024). This is potentially challenging as there might not be as much urgency to try and rectify the root causes of near misses because the incident did not actually occur. By considering the safety examples listed above, it is clear how PSIRF can be applied to perioperative care to learn from safety incidents or near misses, to improve the quality of care provided in what are often high-risk situations.

Although sometimes safety incidents can be unavoidable, it is important that any patient safety incidents and near misses are reported in a timely manner. The reporting of such instances is what allows for learning and improvement of safety. PSIRF emphasises open communication and psychological safety among healthcare professionals and promotion of a learning and blame-free environment, which in turn can decrease near misses and improve patient safety.

A useful tool to aid understanding of what has happened in an incident is the Systems Engineering Initiative for Patient Safety (SEIPS). The SEIPS tool highlights how healthcare processes and outcomes are a product of aspects of the external environment, organisation, internal environment, tools and technology and tasks as well as the person(s) involved. The focus is on what happened and may have gone wrong – and why – rather than who is at fault (Sampson et al 2021), and on informing and supporting staff throughout.

Emerging evidence on the impact of local PSIRF implementation has highlighted that health providers feel better able to identify safety priorities and create improvements in patient care. It will be important to see the findings of national evaluations of PSIRF implementation and how approaches have varied across the country.


Rita Couto Rapid Research Evaluation and Appraisal Lab (RREAL), Department of Targeted Intervention, University College London
John Welch University College London Hospital

